# Reframing Systems Approaches in Psychiatry: Lessons from the Family and Natural Supports (FNS) Model for Youth at Risk of Homelessness

**DOI:** 10.1192/j.eurpsy.2026.11112

**Published:** 2026-07-31

**Authors:** S. Wong, A. David

**Affiliations:** 1 Faculty of Medicine, University of British Columbia, Vancouver; 2 University of Ottawa, Ottawa, Canada

## Abstract

**Introduction:**

Youth at risk of homelessness often face unstable relationships and fragmented care, negatively impacting their mental health. The Family and Natural Supports (FNS) approach reconnects youth with trusted adults, biological or chosen, to build supportive, protective relationships. This model highlights how psychiatrists can extend therapeutic alliances beyond the individual to include key natural supports.

**Objectives:**

To examine the impact of ruptured alliances between youth, FNS, and service providers, and to explore how psychiatrists can reframe systems-based care to better engage youth and their support networks.

**Methods:**

The FNS approach was implemented across nine youth-serving agencies in Canada. Semi-structured interviews were conducted with 28 youth and 19 natural supports (FNS), yielding qualitative data on their experiences. Notably, 12 participants (6 youth and 6 FNS) described a sudden breakdown in communication with their assigned worker. A thematic analysis was conducted to explore the implications of these ruptures.

**Results:**

Youth and FNS participants described emotional consequences of disconnection, including feelings of abandonment, lack of trust for future programs, reduced self-esteem and self-worth, and reduced emotional safety and mental health in both youth and FNS participants.

**Conclusions:**

For psychiatrists working with youth at risk of homelessness, the FNS model challenges conventional individual-centered approaches by emphasizing dual therapeutic alliances: one with the youth and one with their natural supports. These relationships are essential for promoting psychological stability and supporting youths’ transitions into adulthood. The findings underscore the need for psychiatric care systems to intentionally engage youth in the context of their chosen relationships, recognizing these connections as essential to mental health outcomes. Reframing psychiatric care in this way invites a broader, more relationally anchored practice that may improve engagement, reduce dropouts, and promote sustained recovery. Future research should explore how such system-level shifts affect long-term outcomes in youth mental health and housing stability.

**Disclosure of Interest:**

None Declared

